# ECMO Retrieval Program: What Have We Learned So Far

**DOI:** 10.3390/life13010157

**Published:** 2023-01-05

**Authors:** Ihor Krasivskyi, Clara Großmann, Marit Dechow, Ilija Djordjevic, Borko Ivanov, Stephen Gerfer, Walid Bennour, Elmar Kuhn, Anton Sabashnikov, Navid Mader, Kaveh Eghbalzadeh, Thorsten Wahlers

**Affiliations:** 1 Department of Cardiothoracic Surgery, University Hospital Cologne, 50937 Cologne, Germany; 2 Department of Cardiothoracic Surgery, Helios Hospital Siegburg, Heart Centre, 53721 Siegburg, Germany

**Keywords:** ECMO, cardiogenic shock, mortality

## Abstract

Veno-arterial extracorporeal membrane oxygenation (VA-ECMO) is increasingly used for patients with cardiogenic shock or cardiac arrest. However, survival rates remain low. It is unclear to what extent ECMO patients benefit from the ECMO team learning curve. Therefore, we aimed to analyze our mobile ECMO program patients from the past seven years to evaluate if a learning curve benefits patients’ outcomes. We analyzed 111 patients from our databank who were supported with a VA-ECMO and brought to our hospital from January 2015 to December 2021. Patients were divided into two groups: survival (n = 70) and non-survival (n = 41). As expected, complications after ECMO implantation were more severe in the non-survivor group. The incidence of thromboembolic events (*p* = 0.002), hepatic failure (*p* < 0.001), renal failure (*p* = 0.002), dialysis (*p* = 0.002) and systemic inflammatory response syndrome (SIRS, *p* = 0.044) occurred significantly more often compared with the survivor group. We were able to show that despite our extensive experience in terms of ECMO retrieval program the high mortality and morbidity rates stay fairly the same over the years. This displays that we have to focus even more on patient selection and ECMO indication.

## 1. Introduction

Despite recent advances, patients with a cardiac arrest or cardiogenic shock still have a high mortality rate [1]. Veno-arterial extracorporeal membrane oxygenation (VA-ECMO) is increasingly used for those patients to provide temporary circulatory support and improve the hemodynamic status [2]. However, survival rates remain low and the selection of suitable patients continues to prove challenging [3]. A proper evaluation of the patients before implantation is critical in order to reduce mortality and morbidity rates as well as healthcare expenditures. 

There are a large number of recommendations and scoring systems regarding ECMO implantation in patients with cardiogenic shock and cardiac arrest [4]. However, official guidelines in cases of ECMO implantation are rather indefinite [4]. Therefore, the decision for or against ECMO is often made on an individual basis thereby taking factors like age, body mass index (BMI), indication for ECMO and duration of cardiopulmonal resuscitation (CPR) into account [5,6].

ECMO retrieval programs are relatively new but have been on the rise especially in the COVID pandemic with veno-venous (VV) ECMOs [7]. With each year ECMO programs gain knowledge and experience in the treatment of patients. However, it is unclear to what extent ECMO patients benefit from the learning curve of the programs and hospitals. Different studies have shown a minimum patient requirement for acceptable results, but have not described the effect beyond that [8,9].

Therefore, we aimed to analyze patients from our mobile ECMO program from the past seven years to evaluate if a learning curve benefits these patients or if a thorough patient selection is of greater value in terms of mortality. 

## 2. Materials and Methods

This study was designed as a retrospective single center non-randomized study reviewing and analyzing the collected data. The data was gathered in the period from January 2015 to December 2021. All 111 patients, who underwent mobile ECMO therapy (Cardiohelp, Maquet, Rastatt, Germany) were included in our study. They were divided into two groups: survivors (n = 41) and non-survivors (n = 70) and were analyzed retrospectively. Part of the data has been previously described [10].

### 2.1. ECMO-Center Protocol

Our mobile ECMO program and protocol have already been described elsewhere [10,11]. All implantations were performed on-site in tertiary hospitals. Contact was usually initiated by the referring clinic. The ECMO therapy was implemented according to the Extracorporeal Life Support Organization (ELSO) recommendations [4].

### 2.2. Data Collection

All data were received from the institutional database and were analysed retrospectively. We examined patients’ demographic characteristics, status before ECMO support, laboratory parameters and early in-hospital short-term outcome data.

### 2.3. Outcome Analysis

All-cause in-hospital mortality was the primary endpoint in our study. Stroke, bleeding, limb ischemia, acute respiratory distress syndrome (ARDS), dialysis, septic shock and duration (days) of intensive care unit (ICU) stay were secondary endpoints in the present study. 

### 2.4. Ethics

Based on the Declaration of Helsinki (as revised in 2013) and the Ethics Committee of the Medical Faculty of the University of Cologne we were released from an obligation to receive an ethical approval. According to the German law, no additional documents from the local ethics committee are required in order to write purely retrospective clinical research.

### 2.5. Statistical Methods

Continuous variables were examined for normality of distribution. Normally distributed variables were analyzed using Student *t*-Test, whereas a Mann–Whitney U test was used for not normally distributed variables. In order to examine categorical variables, we performed a Chi-square test. We presented all continuous variables as mean ± standard deviation (SD). On the other hand, categorical variables are shown in percentages. We used Fisher exact test indicator for those variables, when the minimum expected count of cells was <5. All analysis were conducted using a *p*-value of <0.05 as significant. We performed linear regressions in order to define the survival trend by years. Survival in different time periods was tested for statistical significance using a weighted least squares regression, assuming a linear trend. Statistical analysis was conducted with the support of Statistical Package for Social Sciences, version 28.1 (SPSS Inc., Chicago, IL, USA).

## 3. Results

### 3.1. Indication of ECMO Therapy

**Figure 1:**
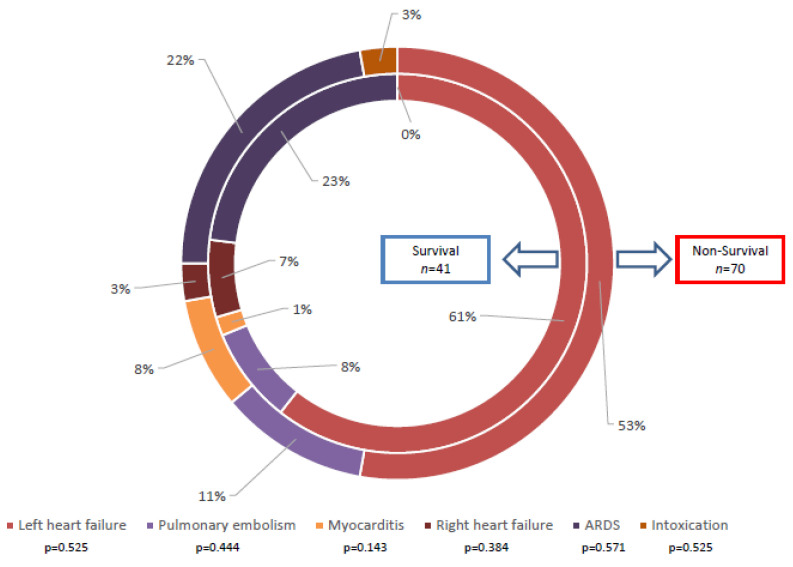
Reasons for ECMO implantation comparing the two groups (survivors (*n* = 41) vs. non-survivors (*n* = 70)).

In total, 111 patients underwent mobile ECMO therapy between January 2015 and December 2021 and were included. Six patients were previously excluded as cannulation was unsuccessful in two patients and four did not survive the transport in our hospital. Cardiogenic shock with left heart failure was the main indication for ECMO therapy (61% survival group vs. 53% non-survival group, Figure 1). Others included pulmonary embolism (11% vs. 8%), myocarditis (8% vs. 1%), right heart failure (3% vs. 7%), acute respiratory distress syndrome (ARDS, 22% vs. 23%) and intoxication (3% in the non-survival group). After the analysis was conducted no statistically significant values regarding ECMO indications between survival and non-survival group were found. 

### 3.2. Demographic and Clinical Characteristics

The demographic and clinical characteristics of the two groups can be seen in Table 1. Patients in both groups were generally young with mean ages of 55 ± 13 and 49 ± 14 years (*p* = 0.719). There were significantly (*p* = 0.027) more male patients in the non-survivor group (75.7%) than in the survivor group (56.1%). In terms of comorbidities the groups were fairly equal and showed an expected range of diseases including cardiovascular risk factors. 

### 3.3. Clinical Characteristics of Implantation

The implantation data can be seen in Table 2. Non-survivors had a significantly longer resuscitation duration than survivors (52 ± 49 min versus 22 ± 24 min, *p* = 0.005). There was no significant difference (*p* = 0.426) in patients receiving an intra-aortal balloon pump (IABP) in addition to ECMO. The number of further support via Impella implantation was higher in survivors (4.6% (non-survivors) versus 17.1% (survivors), *p* = 0.043). Further data did not differ significantly between both groups. 

### 3.4. Laboratory Parameters at Admission and 48 h after ECMO Implantation

The laboratory parameters at admission, which were taken at the referring clinic before ECMO implantation, and 48 h after ECMO implantation can be seen in Table 3 and Table 4. At admission, bilirubin was significantly lower (*p* = 0.002) in survivors compared with non-survivors, whereas CK-MB levels were significantly higher (*p* = 0.012) in the non-survival group. 

After 48 h, CK-MB levels were significantly higher (*p* = 0.020) in the non-survival group compared with the survival group. Moreover, lactate was also significantly higher (*p* = 0.007) in the non-survival group.

### 3.5. Complications after ECMO Implantation

Table 5 shows the complications after the ECMO implantation. There were significantly more thromboembolic events in the non-survivor groups with 33.3% versus 7.3% in the survivor group (*p* = 0.002). Limb ischemia was quite similar with occurrences in 19.0% of non-survivors and 17.1% of survivors (*p* = 0.799). Hepatic failure occurred significantly more often in the non-survivor group (44.4% versus 12.2%, *p* < 0.001). Renal failure and the number of patients requiring dialysis also happened more often in the non-survivor group (*p* = 0.002 and *p* = 0.002, respectively). Patients in the survivor group stayed significantly longer in the intensive care unit with 17 ± 14 days compared with 7 ± 7 days in the non-survivor group (*p* < 0.001). 

### 3.6. Survival Rates for Each Year from 2015 Till 2021

**Figure 2:**
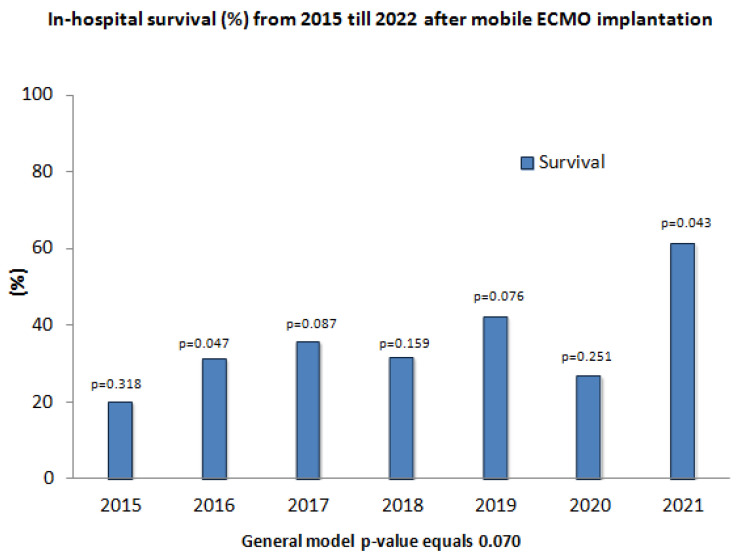
In-hospital survival (%) from 2015 till 2022 after mobile ECMO implantation.

Figure 2 shows the survival rates for each year from 2015 to 2021. It can be seen that the mortality rate was the highest in the first year (2015) with 80.0% and the lowest in the last year (2021) with 38.9%. Despite the statistically significant differences for some years, the general model *p*-value equals 0.070 and was not statistically significant under the 95% of confidence interval (CI).

## 4. Discussion

As already described, VA-ECMO has proven to be a rescue tool in emergency situations and represents a valuable treatment option in order to restore hemodynamics and provide cardiac as well as respiratory support [12]. Our data showed an overall survival rate of 37% for patients with mobile ECMO support, which is comparable to the reported rates for non-mobile ECMO therapy in cardiogenic shock of around 40% [13]. In addition, our data shows high morbidity rates with complications occurring in most patients. 

In order to analyze whether the gain of knowledge and experience over the years benefits ECMO patients we looked at the survival rate for each year. As shown above, the survival rate was the highest for 2021 and the lowest for 2015 which could indicate a better treatment for patients. Scolari et al. [14] have also described an increased survival rate over time possibly due to a learning curve.

In general, the mortality rates were very high in all years even if they were a little lower than in 2015. This could confirm previous findings that centers should treat a certain number of ECMO patients, but after that there is only little benefit to be had for the patients from improved training and experience. Patients requiring ECMO for the indications mentioned above are critically ill and have a high likelihood of death. 

However, as can be seen, the number of additionally implanted Impellas has increased over the last few years. As ECMO during cardial resuscitation is usually placed via the femoral artery and vein, a potential left ventricular overload has been described [15]. Therefore, different studies have looked at further devices to unload the left ventricle such as an Impella [16]. In our study the use of additional Impellas was significantly higher in the survivor group (*p* = 0.043). This agrees with various papers that have shown a positive impact of Impella during ECMO therapy and decreased mortality rates [17]. Even though complications might be elevated with ECMO and Impella (“ECPELLA”), as a recent review showed, the mortality seems to be lower [18,19]. In addition, optimal timing regarding concomitant Impella implantation might play a crucial role in patients’ survival [20]. Moreover, authors have mentioned that sufficient device sizing and convenient patient selection could reduce a complication rate and improve outcomes [21].

The selection of suitable patients is crucial to increase the survival rate and reduce mortality and morbidity. Smith et al. [22] showed that a shorter ECMO run is associated with higher mortality. This could reflect patients who were too sick in the first place or had a diagnosis incompatible with life, for example neurological injuries. Vakil et al. [13] had a slightly higher survival rate than has previously been described in the literature and, overall, a longer ECMO duration. They discussed that this was due to fewer “too sick” patients and showed a better patient selection overall [13]. 

Different predictors of in-hospital mortality have been described, including the duration of cardiopulmonary resuscitation [23]. In our study, the duration was significantly higher (*p* = 0.005) in the non-survivor group than in the survivor group. This demonstrates that patients after CPR should be very carefully evaluated for if they are suitable for ECMO therapy [24]. 

In our study, there was no significant difference (*p* = 0.547) in the distance to the patient between the two groups, which is in accordance with other studies [25]. This indicates that the coordination between the tertiary hospitals and the mobile ECMO team worked well and efficiently. Research has shown that ECMO retrieval programs are safe and provide valuable option [7]. 

Based on our data, the lactate level was not significantly higher (*p* = 0.007) in the non-survivor group than in the survivor group. However, Fux et al. [26] demonstrated that arterial lactate and number of vasopressors were independent predictors of 90-day mortality. This can be explained by a more severe cardiogenic shock in patients with higher lactate levels [26]. In addition, the level of CK-MB at admission as well as after 48 h was significantly higher in non-survivors than in survivors (*p* = 0.012 and *p* = 0.020, respectively). CK-MB has also been described to be associated with higher mortality on ECMO support in patients with postcardiotomy cardiogenic shock [27]. Therefore, our data demonstrate that higher myocardial cell damage indicating a more severe myocardial infarction or shock situation are linked to worse survival rates. These patients might be too sick for ECMO therapy. However, it remains a challenge to evaluate patients in emergency situations where most laboratory markers are not at hand. 

The assessment of patients to be put on ECMO is not only important regarding mortality and morbidity but also healthcare cost [28]. Maxwell et al. [28] described that the cumulative national charges for ECMO in the United States of America have increased drastically. This shows that ECMO therapy is used more frequently but potentially also in the wrong candidates, prolonging survival in patients with diagnoses incompatible with life [28]. 

As previously described, complications in patients with ECMO therapy were high. Thromboembolic events (*p* = 0.002), hepatic failure (*p* ≤ 0.001), renal failure (*p* = 0.002), dialysis (*p* = 0.002) and SIRS (*p* = 0.044) were statistically significantly more frequent in non-survivors. The reported incidence of acute kidney disease during ECMO therapy is around 45% [29]. Renal failure is one of the early signs indicating multiorgan failure which often ends deadly [13,29]. It is not quite clear if it directly increases the risk of mortality or is merely an indicator of the severity of the illness [28]. Limb ischemia was also high in both groups with 19% in non-survivors and 17.1% in survivors. However, there was no statistical difference in our study, whereas Kaushal et al. [5] demonstrated limb ischemia as an independent predictor of mortality. 

Moreover, another study mentioned that platelets count, bilirubin and creatinine levels were predictors of in-hospital mortality after ECMO implantation [30]. In addition, Kohs et al. found that severe thrombocytopenia was associated with adverse outcomes in patients on ECMO [31]. Further authors have shown that serum bilirubin elevation was associated with an increased risk of in-hospital mortality after ECMO [32]. Likewise, Joo et al. mentioned that total bilirubin (*p* = 0.017), acute kidney injury (*p* = 0.005) and dialysis (*p* = 0.001) were predictors for adverse outcomes and were associated with higher mortality in patients on ECMO [33]. In addition, an elevated total bilirubin level (*p* = 0.040) and an elevated lactate level (*p* = 0.002) were risk factors for in-hospital mortality after ECMO initiation [34]. In our study, Bilirubin levels were significantly higher (*p* = 0.002) in the non-survival group compared with the survival group. The creatinine and platelets count were also higher in the non-survival group. All the above-mentioned factors could be potential confounders for the outcomes of our analysis and should be taken into account during the interpretation of our results. 

We were also able to confirm that the number of red blood cells (RBC) as well as other blood products on ECMO support was very high [35]. A mean of 19 ± 16 (non-survivors) and 17 ± 20 (survivors) RBC were transfused on ECMO support [36]. This also shows the severity of the illness of the patients. On the other hand, patients that are were discharged from the hospital have shown a mean survival of 8.1 years and a reasonable quality of life in a study by Cankar et al. [37]. In these cases, the patient benefited from the intensive treatment. 

### Limitations

The statistical power of this study is reduced because of several factors. Firstly, it was designed as retrospective non-randomized research with a limited number of patients from a single center and the sample size calculation was omitted. Secondly, this manuscript presents information regarding only in-hospital short-term outcomes and did not evaluate long-term results and quality of life measures. Thirdly, the survivor group had better renal and hepatic parameters prior to VA-ECMO and that could be a potential confounder for the outcomes. In order to verify this, a prospective larger multicentral studies should be conducted. Fourthly, the authors only had access to the available variables in electronic or written patient notes and flowcharts. Therefore, not all the relevant laboratory markers and related timings could be analyzed.

## 5. Conclusions

In conclusion, we were able to show that, despite our extensive experience in terms of our ECMO retrieval program, the high mortality rates stay fairly similar over the years. This depicts that we have to focus even more on patient selection and evaluating the ECMO indication carefully. Official criteria would be optimal; however, they are hard to manifest as ECMO therapy is often a case-by-case decision and randomized controlled trials are ethically very difficult to implement. 

## Figures and Tables

**Table 1:** Demographic and clinical characteristics (n = 111).

	Non-Survival (n = 70)	Survival (n = 41)	*p*-Value
Age (years), mean ± SD	55 ± 13	49 ± 14	0.719
Male, n (%)	53 (75.7%)	23 (56.1%)	0.027
BMI (kg/m^2^), mean ± SD	28 ± 7	28 ± 6	0.773
EuroSCORE II (%), mean ± SD	6.0 ± 3.6	7.0 ± 3.6	0.849
Previous MI, n (%)	16 (25.8%)	9 (22.0%)	0.419
Previous stroke, n (%)	2 (3.2%)	2 (4.9%)	0.516
COPD, n (%)	8 (12.7%)	3 (7.3%)	0.298
Smoking, n (%)	21 (33.3%)	15 (36.6%)	0.733
Chronic renal insufficiency, n (%)	9 (14.3%)	2 (4.9%)	0.113
Dialysis, n (%)	4 (6.3%)	3 (7.3%)	0.572
Diabetes, n (%)	18 (28.6%)	11 (26.8%)	0.846
Arterial hypertension, n (%)	29 (46.0%)	17 (41.5%)	0.647
Hyperlipidaemia, n (%)	15 (23.8%)	10 (24.4%)	0.946
SARS-CoV-2, n (%)	7 (11.1%)	3 (7.3%)	0.736

MI, myocardial infarction; BMI, body mass index; COPD, chronic obstructive pulmonary disease.

**Table 2:** Implantation data (n = 111).

	Non-Survival (n = 70)	Survival (n = 41)	*p*-Value
ALS before ECMO, n (%)	39 (56.5%)	22 (53.7%)	0.770
Duration ALS, min, mean ± SD	52 ± 49	22 ± 24	0.005
Distance to patient (km), mean ± SD	26 ± 24	27 ± 28	0.547
Implantation technique, PP, n (%)	55 (94.8%)	27 (90.0%)	0.406
Initial ECMO flow, L/m, mean ± SD	3.9 ± 0.8	3.8 ± 0.9	0.638
ECMO duration, hours, mean ± SD	108 ± 110	106 ± 78	0.003
IABP, n (%)	3 (4.6%)	4 (9.8%)	0.426
Impella, n (%)	3 (4.6%)	7 (17.1%)	0.043
ECMO weaning, n (%)	8 (11.9%)	41 (100%)	<0.001
RBC, n, mean ± SD	19 ± 16	17 ± 20	0.552
FFP, n, mean ± SD	10 ± 12	7 ± 12	0.405
Platelets, n, mean ± SD	2 ± 2	2 ± 4	0.355

ECMO, extracorporeal membrane oxygenation; IABP, intra-aortic balloon pump; Impella, CP*^®^*, circulatory support device; ALS, advanced life support; RBC, red blood cell; FFP, fresh frozen plasma; PP, per punktura.

**Table 3:** Laboratory parameters before ECMO implantation (n = 111).

	Non-Survival (n = 70)	Survival (n = 41)	*p*-Value
Hb (g/dL), mean ± SD	9.1 ± 10.8	7.7 ± 1.4	0.221
RBC (10^9^/L), mean ± SD	2.6 ± 0.5	2.7 ± 0.5	0.961
WBC (10^9^/L), mean ± SD	21.7 ± 9.9	22.0 ± 8.7	0.218
Platelets (10^9^/L), mean ± SD	54.2 ± 48.8	98.8 ± 129.6	0.065
Bilirubin, mg/dL, mean ± SD	8.2 ± 11.3	4.2 ± 7.2	0.002
AST (U/L) max, mean ± SD	3535 ± 4322	2388 ± 4516	0.557
ALT (U/L) max, mean ± SD	1350 ± 1595	1252 ± 2212	0.148
Creatinine (mg/dL), mean ± SD	2.2 ± 1.7	2.5 ± 4.1	0.140
Lactate (mmol/L), mean ± SD	9.7 ± 6.7	6.6 ± 4.9	0.098
CK, U/L, mean ± SD	2921 ± 3730	3868 ± 1391	0.131
CK-MB, U/L, mean ± SD	336 ± 362	167 ± 220	0.012

CK, creatine kinase; CK-MB, creatine kinase MB; AST, aspartate transaminase; ALT, alanine transaminase; Hb, hemoglobin; WBC, white blood cell; RBC, red blood cell.

**Table 4:** Laboratory parameters after ECMO implantation (48 h) (n = 111).

	Non-Survival (n = 70)	Survival (n = 41)	*p*-Value
pO2 (mmHg), mean ± SD	130 ± 65	122 ± 45	0.085
pCO2 (mmHg), mean ± SD	39 ± 5	37 ± 6	0.201
pH, mean ± SD	7.4 ± 0.07	7.4 ± 0.07	0.478
FiO2 (%), mean ± SD	55 ± 26	68 ± 51	0.052
CK, U/L, mean ± SD	3377 ± 5736	2725 ± 7002	0.713
CK-MB, U/L, mean ± SD	126 ± 119	71 ± 92	0.020
Creatinine (mg/dL), mean ± SD	2.7 ± 3.4	2.2 ± 1.8	0.487
Lactate (mmol/L), mean ± SD	5.2 ± 5.3	2.9 ± 2.4	0.007
Bilirubin (mg/dL), mean ± SD	2.7 ± 3.7	2.4 ± 2.5	0.376
AST (U/L), mean ± SD	2395 ± 3388	1604 ± 3846	0.502
ALT (U/L), mean ± SD	915 ± 1339	917 ± 1844	0.207
aPTT (s), mean ± SD	64 ± 27	60 ± 28	0.657

pO2, oxygen partial pressure; pCO2, carbon dioxide partial pressure; pH, potential of hydrogen; FiO2, fraction of inspired oxygen; AST, aspartate transaminase; ALT, alanine transaminase; aPTT, partial thromboplastin time; CK, creatine kinase; CK-MB, creatine kinase MB.

**Table 5:** Complications after ECMO implantation (n = 111).

	Non-Survival (n = 70)	Survival (n = 41)	*p*-Value
Stroke, n (%)	9 (14.3%)	2 (5.0%)	0.195
Thromboembolic events, n (%)	21 (33.3%)	3 (7.3%)	0.002
Bleeding, n (%)	31 (51.7%)	14 (35.0%)	0.101
Limb ischemia, n (%)	12 (19.0%)	7 (17.1%)	0.799
Limb ischemia requiring intervention, n (%)	6 (9.5%)	5 (12.2%)	0.749
ARDS, n (%)	19 (30.6%)	7 (17.5%)	0.137
Pneumonia, n (%)	19 (30.6%)	14 (35.0%)	0.646
Pneumothorax, n (%)	5 (8.1%)	2 (5.0%)	0.702
Hepatic failure, n (%)	28 (44.4%)	5 (12.2%)	<0.001
Gastrointestinal bleeding, n (%)	4 (6.5%)	1 (2.4%)	0.646
Ventricular rhythm disorders, n (%)	21 (33.9%)	16 (39.0%)	0.594
PPI, n (%)	1 (1.6%)	3 (7.3%)	0.299
Renal failure, n (%)	45 (71.4%)	16 (39.0%)	0.002
Dialysis, n (%)	31 (50.0%)	8 (19.5%)	0.002
Oxygenator failure, n (%)	2 (3.0%)	0 (0.0%)	0.526
Wound infection, n (%)	2 (3.2%)	5 (12.2%)	0.112
SIRS, n (%)	25 (39.7%)	8 (20.5%)	0.044
Septic shock, n (%)	25 (39.7%)	9 (22.5%)	0.071
ICU stay (days), mean ± SD	7 ± 7	17 ± 14	<0.001

ICU, intensive care unit; SIRS, systemic inflammatory response syndrome, PPI, permanent pacemaker implantation; ARDS, acute respiratory distress syndrome.

## Data Availability

Data is available on a special request.
